# An Electrochemical Study of the Effect of Sulfate on the Surface Oxidation of Pyrite

**DOI:** 10.3390/ma17215145

**Published:** 2024-10-22

**Authors:** Siqi Lv, Yujian Liang, Xuezhen Zhang, Xiaomei Tan, Zuotan Huang, Xuan Guan, Chongmin Liu, Zhihong Tu

**Affiliations:** 1 College of Environmental Science and Engineering, Guilin University of Technology, Guilin 541004, China; 19111011262@163.com (S.L.); liangyujian@glut.edu.cn (Y.L.); zxz19980129@163.com (X.Z.); 18290163007@163.com (X.T.); hzt001201@163.com (Z.H.); guanxuan0426@163.com (X.G.); chongmin405@163.com (C.L.); 2 Guangxi Key Laboratory of Environmental Pollution Control Theory and Technology for Science and Education Combined with Science and Technology Innovation Base, Guilin University of Technology, Guilin 541004, China; 3 Collaborative Innovation Center for Water Pollution Control and Water Safety in Karst Area, Guilin University of Technology, Guilin 541004, China; 4 Key Laboratory of Carbon Emission and Pollutant Collaborative Control (Guilin University of Technology), Education Department of Guangxi Zhuang Autonomous Region, Nanning 530021, China

**Keywords:** pyrite, sulfate, electrochemical oxidation, CV, EIS, Tafel polarization curves

## Abstract

Pyrite is one of the most abundant metal sulfide tailings and is susceptible to oxidation, yielding acidic mine drainage (AMD) that poses significant environmental risks. Consequently, the exploration of pyrite surface oxidation and the kinetic influencing factors remains a pivotal research area. Despite the oxidation of pyrite producing a significant amount of sulfate (SO_4_^2−^), a comprehensive investigation into its influence on the oxidation process is lacking. Leveraging pyrite’s semiconducting nature and the electrochemical intricacies of its surface oxidation, this study employs electrochemical techniques—cyclic voltammetry (CV), Tafel polarization, and electrochemical impedance spectroscopy (EIS)—to assess the effect of SO_4_^2^⁻ on pyrite surface oxidation. The CV curve shows that SO_4_^2−^ does not change the fundamental surface oxidation mechanism of pyrite, but its redox peak current density decreases with the increase in SO_4_^2−^, and the surface oxidation rate of pyrite decreases. The possible reason is attributed to SO_4_^2−^ adsorption onto pyrite surfaces, blocking active sites and impeding the oxidation process. Furthermore, Tafel polarization curves indicate an augmentation in polarization resistance with elevated SO_4_^2−^ concentrations, signifying heightened difficulty in pyrite surface reactions. EIS analysis underscores an increase in Weber diffusion resistance with increasing SO_4_^2^⁻, indicating that the diffusion of Fe^3+^ to the pyrite surface and the diffusion of oxidized products to the solution becomes more difficult. These findings will improve our understanding of the influence of SO_4_^2−^ on pyrite oxidation and have important implications for deepening the understanding of surface oxidation of pyrite in the natural environment.

## 1. Introduction

Pyrite, a prevalent metal sulfide found in Earth’s crust, is easily oxidized to produce acid mine drainage (AMD), which leads to serious environmental pollution problems [1,2]. Therefore, many researchers have developed lots of passivation techniques to try to slow down the surface oxidation of pyrite to reduce the generation of AMD [3]. In addition, pyrite is usually accompanied by some precious metals such as Au, Cu, Ag, Ti, etc., in structures [4,5]. In this respect, the mineral metallurgy industry hopes to increase the oxidation rate of the pyrite surface so as to efficiently recover associated precious metals from pyrite structures. Therefore, the mechanism and kinetic influence factors of the pyrite surface oxidation mechanism have always been a research hotspot. Numerous studies have found that the surface oxidation rate of pyrite is closely related to many factors. For example, a large number of studies have indicated that Fe^3+^ [6,7,8], O_2_ [9,10], pH [11,12], microorganisms [13,14], and temperature [9] can significantly affect the surface oxidation rate of pyrite.

In comparison, research on the influences of inorganic ions on the oxidation rate of pyrite is relatively scarce, and the few relevant studies are focused on the influence of Cl^−^. Given that pyrite possesses natural semiconducting properties, its surface oxidation is frequently characterized as an electrochemical process [15,16]. On the basis that electrochemical technology can reflect the subtle changes caused by a certain factor through the in situ electrochemical signal, which is relatively efficient and convenient, electrochemical techniques have proven to be effective tools in exploring the kinetics and underlying mechanisms of pyrite surface oxidation. For instance, Senanayak [17] systematically studied the effect of Cl^−^ on the surface oxidation of pyrite by using an electrochemical method and pointed out that Cl^−^ could accelerate the oxidation rate of pyrite for the following two reasons. Firstly, Cl^−^ can fuse with some metal ions to improve the solubility of some surface oxidation products, which is more conducive to oxidation. Secondly, Cl^−^ also can increase pyrite’s oxidation rate by inhibiting or reducing the formation of the surface passivating elemental substance sulfur (S^0^) [18,19]. However, the results of [20] showed that Cl^−^ could significantly inhibit the surface oxidation of pyrite. Research by Lin [21] indicated that the surface oxidation of pyrite by Cl^−^ is complex: when the anode voltage is 0.60 V (vs. SCE), increasing the concentration of Cl^−^ can inhibit the S^0^ covering the surface of the pyrite, thus significantly speeding up the oxidation rate of pyrite; when the anode voltage is 0.8~1 V (vs. SCE) and the concentration of Cl^−^ is increased, a large amount of Cl^−^ is adsorbed on the electrode surface, which is not conducive to the interface interaction between pyrite and H_2_O and thus slows down the oxidation rate of pyrite. Furthermore, it also showed that the open-circuit potential (E_ocp_) of pyrite decreases with the increase in Cl^−^, which is consistent with the previous study by Moslemi [22]. In contrast, Antonijević [23] used an electrochemical method and compared the effects of four inorganic acids, H_2_SO_4_, HCl, H_3_PO_4_, and HClO_4_, on the surface oxidation of pyrite. The findings indicated that as the concentration of Cl^−^ increases, the E_ocp_ of pyrite also experiences an elevation.

In fact, in the environment, with the continuous oxidation reaction on the surface of pyrite, the amount of SO_4_^2−^ in its surrounding environment will gradually increase, and for instance, its content will be much higher than that of Cl^−^. Therefore, it is of more practical significance to explore the influence of SO_4_^2−^ on the surface oxidation of pyrite. Unfortunately, there is still a lack of systematic research and demonstration on the effect of sulfate on the surface oxidation of pyrite. Therefore, this study employs electrochemical methods (cyclic voltammetry, Tafel polarization, and AC impedance) to explore the effect of sulfate on the oxidation of pyrite surfaces.

## 2. Materials and Methods

### 2.1. Materials

The pyrite samples were taken from Dabaoshan polymetallic sulfide mine (Shaoguan, Guangdong Province, China). Na_2_SO_4_, HCl, and FeCl_3_ are chemical analytical grade, purchased from Shanghai Aladdin Ltd. (Shanghai, China).

### 2.2. Preparation of Pyrite Working Electrode

After selecting the massive pyrite and cutting it into a cuboid with a size of 1 × 1 × 0.5 cm^3^, it was installed in a specially made electrode casing with a surface area of 1 cm^2^ exposed to the electrolyte. The pyrite was connected to the electrode copper wire through a small threaded hole on the side of the electrode casing. To obtain a fresh and shiny pyrite electrode surface, before the electrochemical test, the pyrite bulk electrode was polished with Al_2_O_3_ polishing powder with particle sizes of 9, 3, and 1 μm in sequence, then cleaned with distilled water and acetone to remove oxide products from the electrode surface as much as possible. Finally, the electrode was blown dry with high-purity nitrogen for electrochemical testing. Before the electrochemical measurement, a scanning electron microscope with an energy dispersive spectrometer (SEM-EDS) (Merlin-3700, Germany ZEISS, Oberkochen, Germany) was used to observe and measure the surface morphology and composition of the electrode.

### 2.3. Experimental Procedures

**Figure 1:**
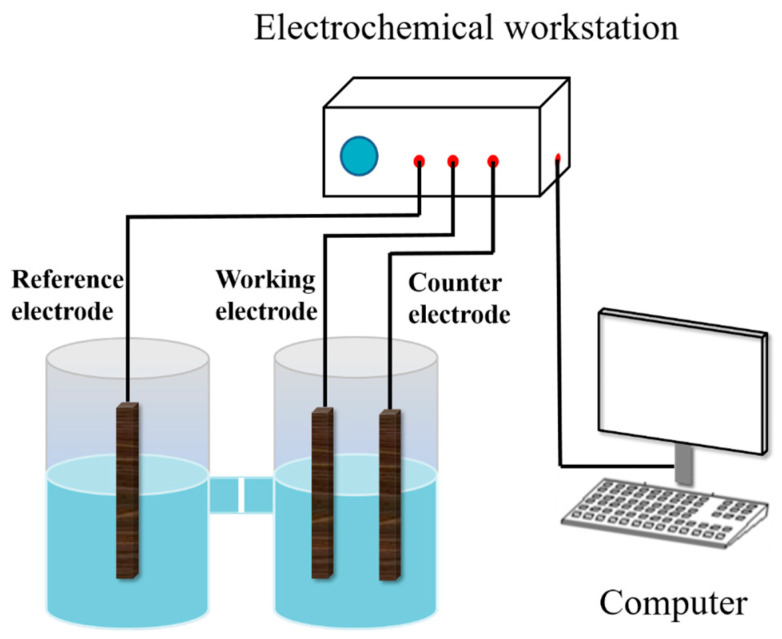
Schematic of electrochemical measuring device.

An H-type three-compartment electrolytic cell was equipped with the pyrite carbon paste electrode as the working electrode, a platinum foil as the counter electrode, and a saturated calomel reference electrode (SCE) with a Luggin capillary salt bridge to minimize the voltage drop across the working and counter electrodes. Unless otherwise stated, all potentials in this work were referenced to the SCE (0.245 V vs. SHE at 25 °C). A simplified schematic diagram of the electrochemical setup used in the experiment is shown in Figure 1. All electrochemical measurements were performed at room temperature with a CHI660 Electrochemical Workstation (CH Instruments, Chenhua Co., Shanghai, China) coupled to a personal computer. The effect of various concentrations of SO_4_^2−^ on the electrochemical oxidation rate of pyrite surfaces was investigated by adding different masses of Na_2_SO_4_ to the control group electrolyte. In addition, the pH of all electrolytes was adjusted to 2 by using HCl, and the process was carried out at 30 °C.

### 2.4. Analytical Methods

In order to ensure the reproducibility and stability of the electrochemical measurements, the pyrite electrode was immersed in the electrolyte for 30 min before each measurement. In this work, cyclic voltammetry, Tafel polarization, and electrochemical impedance spectroscopy were utilized to investigate the influence of SO_4_^2−^ on the electrochemical oxidation of pyrite. In addition, the open-circuit potential (E_ocp_) of the pyrite electrode was also measured before conducting electrochemical tests. The E_ocp_ test lasted 20 min, and the data collection interval was 0.1 s. The CV analysis began scanning at the E_ocp_, with a voltage scan range from −0.60 to 0.80 V, a scan rate of 0.05 V s⁻^1^, two scan cycles, and a sensitivity setting of 1 × 10⁻^3^ A V⁻^1^. The Tafel polarization curve voltage measurement range was from 0.20 V to 0.60 V, and the scanning rate was 5 mV/s. The EIS measurements were also conducted at the potentials of the E_ocp_ with an amplitude of 5 mV and a test frequency range of 0.1~10,000 Hz. The EIS data obtained from the experiment were fitted by the software ZSimpwin3.20 (2004).

## 3. Results and Discussion

### 3.1. Characterization

**Figure 2:**
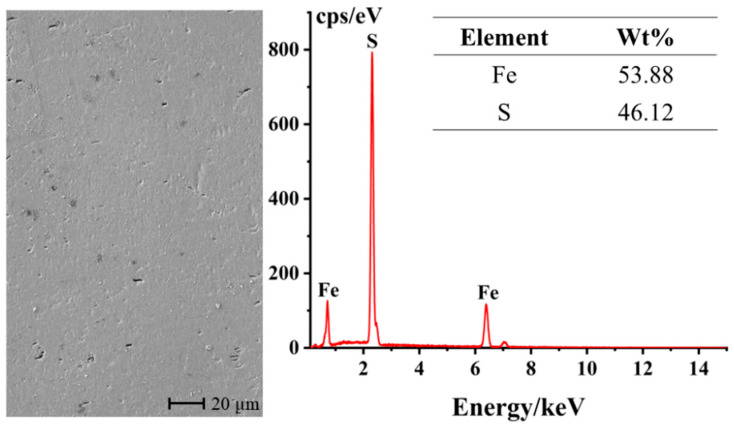
SEM-EDS characterization of the surface of the polished pyrite electrode.

The surface characterization of the unreacted pyrite bulk electrode was conducted using an SEM-EDS. As can be seen from Figure 2, after polishing and grinding, the surface of the pyrite bulk electrode is generally smooth and flat. The few black spots distributed on the electrode surface may be attributed to the presence of minor impurities or lattice defects on the surface. Furthermore, the elemental content analysis reveals that the electrode contains 53.88% sulfur (S) and 46.12% iron (Fe), with an atomic ratio of approximately 2:1, which is very close to the ideal composition of pyrite. This result indicates that the bulk pyrite used in the experiment is of high purity and can meet the requirements of an ideal material for research electrodes.

### 3.2. Open-Circuit Potential (E_ocp_)

The E_ocp_ was established on the pyrite electrode that was not polarized with current from an outer circuit and which was also used to evaluate and feedback the stability of the electrochemical test system. Usually, when the E_ocp_ value fluctuates in a small range, the electrode is in a quasi-steady state in the test system and can well meet the electrochemical test requirements.

**Figure 3:**
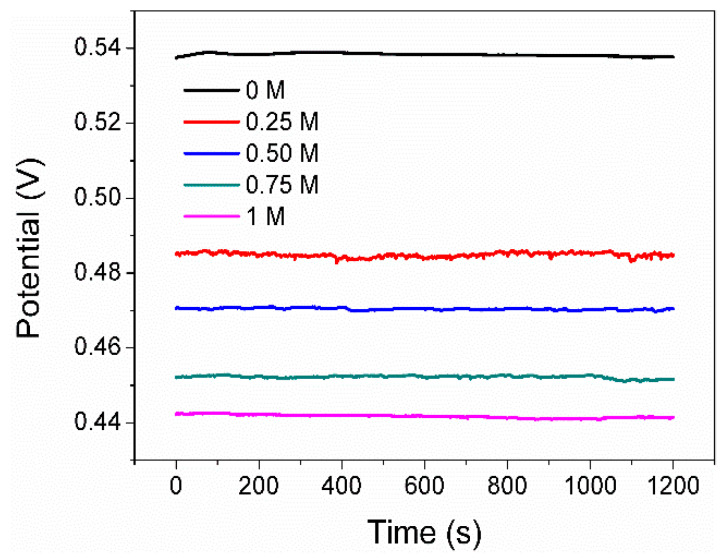
E_ocp_ of pyrite electrode in electrolytes with various concentrations of SO_4_^2−^.

Figure 3 shows the E_ocp_ curves of the pyrite electrode in the solutions with different Na_2_SO_4_ concentrations (0, 0.25, 0.5, 0.75, and 1 mol/L) as a function of time. The value of the E_ocp_ is 0.537V in 0 M SO_4_^2−^. Since the electrolyte contains 1g L^−1^ FeCl_3_, some of the Fe^3+^ adsorbs onto the surface of the pyrite electrode, leading to an increase in the potential of the electrode itself. On the other hand, the abundant Fe^3+^ in the electrolyte elevates the redox potential of the solution itself. Both factors ultimately result in the E_ocp_ of pyrite in this system being much higher than 0.35 V (Ph = 2, and without Fe^3+^, E_ocp_ is approximately 0.35 V). It is also clear that the E_ocp_ is decreased by increasing the concentration of SO_4_^2−^. The gradual decrease in the E_ocp_ upon adding SO_4_^2−^ could be attributed to two main reasons. Firstly, some SO_4_^2−^ readily competes with Fe^3+^ for adsorption on the electrode surface, reducing the amount of Fe^3+^ adsorbed on the electrode and resulting in the oxidation potential of the electrode decreasing [19,20]. Secondly, SO_4_^2−^ ions have the ability to combine with available iron ions present on the pyrite surface, forming complexes and compounds that subsequently lead to a decrease in the E_ocp_. In line with this, previous research has documented that the E_ocp_ of a mineral or metal electrode immersed in solutions containing its metal complexes or compounds is lower compared to that of an electrode in solutions solely comprising its simple metal ions [23].

### 3.3. Cyclic Voltammetry (CV)

**Figure 4:**
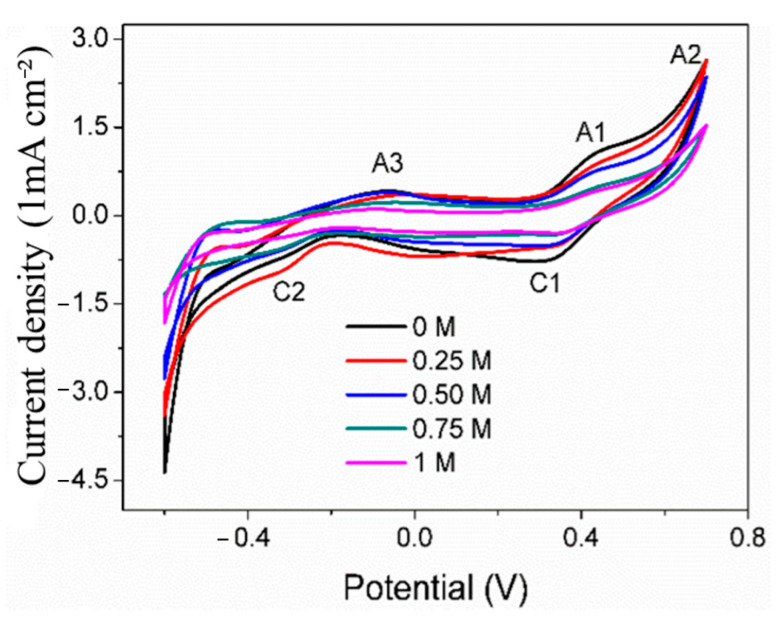
CV curves of pyrite electrode in electrolytes with different concentrations of SO_4_^2−^.

Cyclic voltammetry is usually used to explore the surface oxidation mechanism of the working electrode. Figure 4 shows the CV curves of the pyrite electrode in the electrolyte with the SO_4_^2−^ concentration of 0–1 mol/L. There are three obvious anode peaks on the curves, which are denoted as A1, A2, and A3 (at about 0.50 V, 0.70 V, and −0.1 V, respectively). On the return scan of the pyrite electrode, two cathode peaks were observed at 0.40 V and −0.35 V (denoted as C1 and C2). The above results are in agreement with previous studies [24,25].

The anodic peak A1 is mainly attributed to the reaction in (1), leading to the formation of S_L_ (sulfur-rich layer) and Fe(OH)_3_ [7]. Among them, the sulfur-rich layer is the main component of the sulfur elemental substance (S^0^), accompanied by a small amount of iron-deficient sulfide (Fe_1−x_S_2_) and polysulfide (S_n_^2−^) [26]. When scanning at 0.70 V, a distinct sharp oxidation peak A2 appears, primarily due to the higher oxidation reaction rate of pyrite under this high voltage. At this voltage, the dissolution reaction of pyrite’s main structure forms Fe(OH)_3_ and SO_4_^2−^ (Equation (2)). Secondly, the surface oxidation product S_L_ would be oxidated to SO_4_^2−^ (Equation (3)) [27,28]. The reduction peak C1 might be attributed to the reaction of S^0^ being reduced to H_2_S under this low voltage (Equation (4)).

FeS_2_ + 3H_2_O → Fe(OH)_3_ + 2S_L_ + 3H^+^ + 3e^−^ (1)

FeS_2_ + 11H_2_O → Fe(OH)_3_ + 2SO_4_^2−^ + 19H^+^ + 15e^−^ (2)

S_L_ + 4H_2_O → SO_4_^2−^ + 8H^+^ + 6e^−^ (3)

S^0^ + 2H^+^ + 2e^−^ →H_2_S (4)

The reduction peak of C1 that appears when the pyrite bulk electrode is cathodically scanned in reverse from 0.70 V to 0.40 V may represent the reduction of the previous oxidation product Fe(OH)_3_ to Fe^2+^ (Equation (5)). Continuing the negative scan at −0.35 V, the emergence of peak C2 could be attributed to the occurrence of the following two reduction reactions. Firstly, pyrite is directly reduced to form H_2_S and a substance similar to ferrous sulfide (Equation (6)) [29,30]. Secondly, the reduction reaction of S^0^ on the surface generates H_2_S (Equation (4)) [30,31].

Fe(OH)_3_ + 3H^+^ + e^−^ → Fe^2+^ + 3H_2_O (5)

FeS_2_ + 2H^+^ + 2e^−^ → FeS + H_2_S (6)

When the voltage is scanned anodically in reverse from −0.60 V to −0.10 V, a quite weak anodic peak A3 appears, which is mainly caused by the reverse reaction of (4), where H_2_S is oxidized to S^0^. The reason for this weak oxidation peak lies in the fact that the initially generated H_2_S is not abundant, and furthermore, it is highly insoluble in water. Consequently, it tends to detach from the electrode surface and migrate to the solution interface, ultimately resulting in a very low concentration of H_2_S on the electrode surface.

The slight differences in these curves are mainly reflected in the peak potential and peak current density of some redox peaks. The results show that SO_4_^2−^ does not change the surface oxidation mechanism of pyrite but will certainly affect the surface oxidation reaction rate of pyrite. It can also be seen that with the increase in SO_4_^2−^ concentration, the peak currents of each anode and cathode generally show a tendency to decrease, which reflects that the surface oxidation reaction rate of pyrite decreases with the increase in SO_4_^2−^ concentration. The possible reasons may be due to the SO_4_^2−^ adsorbing on the surface of the pyrite electrode, occupying part of the active surface, which is not conducive to Fe^3+^ oxidation of pyrite. Similarly, previous studies [19] have investigated the oxidation of pyrite electrodes under the conditions of HClO_4_, HCl, and H_2_SO_4_. The results also show that the rate of pyrite is the slowest in the medium containing H_2_SO_4,_ and it is believed that SO_4_^2−^ has the strongest adsorption on the surface of pyrite. Therefore, a large amount of SO_4_^2−^ is adsorbed on the surface of pyrite, which is not beneficial for the oxidation of pyrite by oxidants such as Fe^3+^ to a certain extent.

### 3.4. Tafel Polarization Curve

**Figure 5:**
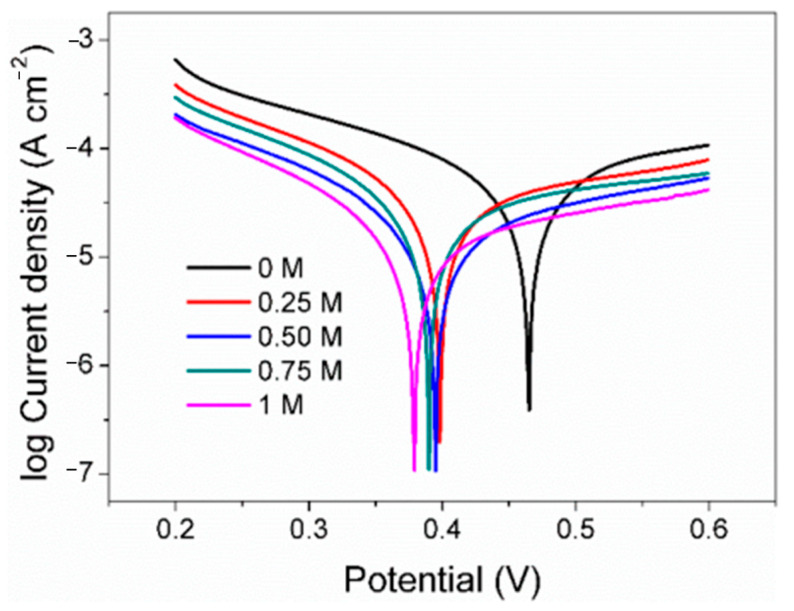
Tafel curve of pyrite electrode in electrolytes with different concentrations of SO_4_^2−^.

As a classical electrochemical method, Tafel polarization is usually used to explore the corrosion rate of electrodes in a certain environment. It is evident from the observations that the pyrite electrode exhibited consistent polarization profiles across all tested conditions, indicative of a uniform electrochemical interaction mechanism occurring despite varying concentrations of SO_4_^2−^ (Figure 5). According to Tafel polarization theory, the corrosion potential (*E*_corr_), corrosion current density (*j*_corr_), and Tafel slopes of the anode (*Β*_a_) and cathode (*β*_c_) were calculated (Table 1).

The *E*_corr_ decreases with increasing SO_4_^2−^, which may be due to the adsorption of more negatively charged SO_4_^2−^ on the electrode surface. In addition, the density of the linear polarization resistance (*R*_L_) also increases with the increase in the concentration of SO_4_^2−^, from the original 760 Ω cm^−2^ which gradually rose to 2044 Ω cm^−2^. Generally, the higher the electrolyte salt content, the faster the electron transfer speed, the more conducive to the reaction, and the smaller the resistance density. However, the results show that the resistance density increases with the increase in SO_4_^2−^. A possible reason for this is that jarosite substances were formed between Fe^3+^ and SO_4_^2−^ on the electrode surface, which slows down the rate of surface oxidation. Corrosion current density in Tafel polarization is widely used to evaluate and reflect the corrosion rate of metal in the theory of metal corrosion. The *j*_corr_ in the control group was 8.67 × 10^−5^ A cm^−2^ and was gradually reduced to 2.28 × 10^−5^ A cm^−2^ with the rise in the concentrations of SO_4_^2−^. The results also indicate that the surface oxidation rate of the pyrite electrode decreases with the increase in SO_4_^2−^. According to the corrosion current density, when the concentration of SO_4_^2−^ in the electrolyte is 0.25 M, 0.50 M, 0.75 M, and 1 M, the surface oxidation rate of pyrite is only 52%, 43%, 28%, and 26% of the blank group, respectively.

According to the classical Tafel polarization curve theory, the slope of the cathode and the slope of the anode can be calculated by Formulas (7) and (8), respectively, where R is the gas constant, T is the temperature, F is the Faraday constant, and *nα* and *nβ* are the anode and cathode electron transfer coefficients, respectively. It can be seen that under the same experimental conditions, the slope of the Tafel polarization curve mainly depends on the electron transfer coefficient. The higher the value, the faster the electron transfer rate and the lower the slope. In this study, both the cathode slope and the anode slope of the Tafel polarization curve gradually increase with the increase in SO_4_^2−^ concentration, indicating that the increase in SO_4_^2−^ concentration leads to the decrease in the electron transfer coefficient, which is not conducive to electron transfer on the electrode surface and finally reflects the gradual decrease in the reaction rate on the surface of pyrite.

(7) $$B_{a}=\frac{2.303RT}{n\alpha F}$$

(8) $$\beta_{c}=\frac{2.303RT}{n\beta F}$$

### 3.5. Electrochemical Impedance Spectroscopy (EIS)

**Figure 6:**
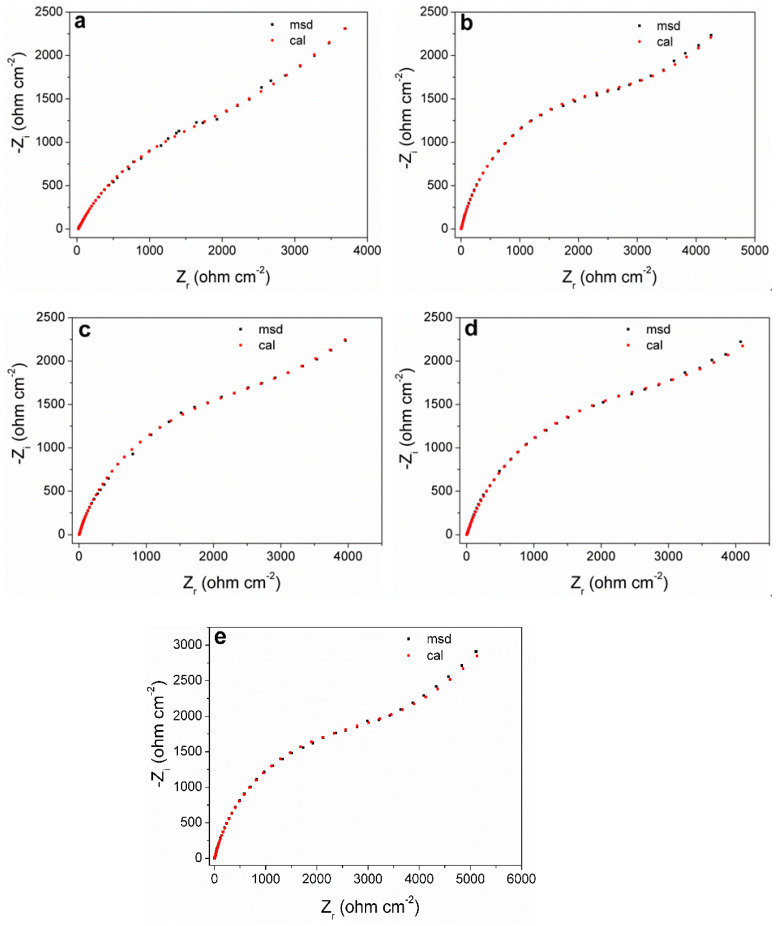
Experimental and simulated Nyquist plots of pyrite electrode in electrolytes with different concentrations of SO_4_^2−^: (**a**) 0 M; (**b**) 0.25 M; (**c**) 0.50 M; (**d**) 0.75 M; (**e**) 1 M.

Figure 6 shows the Nyquist plots of the measured and fitted data obtained by equivalent circuits for the pyrite electrode under different concentrations of SO_4_^2−^. It can be seen that the shape of the EIS curves (black dots) at different concentrations of SO_4_^2−^ do not show significant differences, which also reflects that SO_4_^2−^ will not significantly change the oxidation mechanism of the pyrite surface.

**Figure 7:**
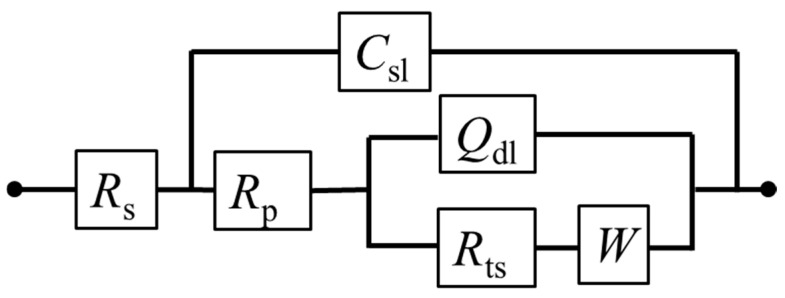
Equivalent electrical circuits proposed for fitting EIS curves of pyrite electrode in electrolytes.

According to the EIS curve and the characteristics of the electrochemical process of the pyrite surface, the equivalent circuit *R*_s_(*C*_sl_(*R*_p_(*Q*_dl_(*R*_ts_*W*)))) can be proposed to fit the EIS curves (Figure 7). Among them, the physical significance of each electrochemical element in the fitting circuit is as follows: *C*_sl_ is the interface capacitance formed between the surface of the pyrite electrode and electrolyte solution; *R*_s_ and *R*_p_ are the resistance generated by solution resistance and interface capacitance (*C*_sl_), respectively; *Q*_dl_ is the double capacitor layer formed between Heimholtz and the electrode surface layer; *R*_ts_ represents the charge transfer resistance; *W* is the Warburg diffusion impedance. In fact, the curve fitted by the circuit (red dot) has a high degree of agreement with the curve measured by the experiment (black dots), which reflects that the fitting circuit used can indeed be relatively close to the kinetic process of pyrite in the electrolyte.

The numerical values of each electronic component in the fitting circuit are summarized in Table 2. It was noted that the solution resistance density *R*_s_ decreased with the increase in SO_4_^2−^ concentration. The reason is that with a higher concentration of electrolyte, the conductivity of the electrolyte will inevitably increase. The trend of *W* gradually increased, indicating that the increase in SO_4_^2−^ concentration was not conducive to the mass transfer process of the electrode reaction, which may be attributed to the diffusion resistance of Fe^3+^ to the pyrite electrode surface and/or that of the reaction product on the electrode surface to the solution increased. Futhermore, the transfer resistance *R*_ts_ also increased gradually. When SO_4_^2−^ was 1 M, *R*_ts_ increased to 3732 Ω cm^−2^, which also reflects that the reaction rate of electrode surface oxidation slowed down with the increase in SO_4_^2−^. One possible reason is that a small amount of SO_4_^2−^ attached to the surface of the electrode, leading to the blocked electron transfer.

## 4. Conclusions

A large amount of SO_4_^2−^ will inevitably be formed during the oxidation process on the surface of pyrite. However, few reports on whether SO_4_^2−^ will affect the surface oxidation of pyrite exist. Pyrite is a semiconductor, so the microscopic effect of environmental factors on its surface oxidation can be explored with the help of classical electrochemical methods. In this study, the E_ocp_, CV, Tafel polarization curves, and EIS electrochemical techniques were used to explore the influence of SO_4_^2−^ on the electrochemical oxidation rate of the pyrite surface, and the following conclusions were obtained: (1) The E_ocp_ and E_corr_ of pyrite decreased with the increase in SO_4_^2−^. (2) The CV curve and EIS curve showed that SO_4_^2−^ did not change the electrochemical oxidation mechanism of the pyrite surface, but the increase in SO_4_^2−^ concentration increased the electron transfer resistance and thus reduced the oxidation rate. (3) SO_4_^2−^ can easily compete with Fe^3+^ on the surface of the electrode, reducing the adsorption of Fe^3+^ on the electrode and thus reducing the oxidation of Fe^3+^ on the surface of pyrite.

## Figures and Tables

**Table 1:** Tafel parameters of pyrite electrode in electrolytes with different concentrations of SO_4_^2−^.

SO_4_^2−^ (mol/L)	*E*_corr_ (V)	*R*_L_ (Ω cm^−2^)	*β*_c_ (mV decade^−1^)	*Β*_a_ (mV decade^−1^)	*j*_corr_ (10^−5^ A cm^−2^)
0	0.465	760.3	117.8	114.9	8.67
0.25	0.398	1057.2	151.51	176.7	4.52
0.50	0.395	1323.4	180.6	219.4	3.75
0.75	0.390	2044.6	210.7	294.3	2.46
1	0.379	2298.8	245.6	324.1	2.28

**Table 2:** The parameters of the equivalent circuit simulated from the EIS curves.

SO_4_^2−^(mol/L)	0	0.25	0.50	0.75	1
*R*_s_ (Ω cm^2^)	20.67	3.69	2.75	2.73	2.34
*C*_sl_ (10^−7^ F cm^−2^)	2.17	1.81	1.75	1.52	1.03
*R*_p_ (Ω cm^2^)	23.87	3.08	5.31	5.82	6.74
*R*_ct_ (kΩ cm^−2^)	3043	3326	3450	3694	3732
*W* (10^−4^ S s^0.5^ cm^−2^)	4.07	4.93	4.99	5.32	5.94

## Data Availability

The original contributions presented in the study are included in the article, further inquiries can be directed to the corresponding author.
